# Prediction of Self-Perception of Patient in Rheumatoid Arthritis With the Key RNAs Expression Profiles

**DOI:** 10.3389/fmed.2020.00567

**Published:** 2020-09-18

**Authors:** Jianting Wen, Jian Liu, Bing Wang, Hui Jiang, Lei Wan, Ling Xin, Yue Sun, Yanqiu Sun, Ying Zhang, Xinlei Du, Xin Wang, Jie Wang

**Affiliations:** ^1^Anhui University of Traditional Chinese Medicine, Hefei, China; ^2^Department of Rheumatology and Immunology, First Affiliated Hospital of Anhui University of Traditional Chinese Medicine, Hefei, China; ^3^Institute of Rheumatology, Anhui Academy of Traditional Chinese Medicine, Hefei, China; ^4^School of Electrical and Information, Anhui University of Technology, Ma'anshan, China

**Keywords:** RNAs, rheumatoid arthritis, self-perception of patient, RNA, logistic-regression analysis, spearman correlation analysis

## Abstract

Self-perception in patients is their self-response to sensory stimuli. It is an important aspect of the existence and quality of life among patients. However, the inherent relationship between self-perception and the cellular activity at the molecular level is elusive. In this study, we aimed to explore the association of self-perception with RNA expression profile in patients with rheumatoid arthritis (RA) through computational analysis. We recruited 30 patients clinically diagnosed with RA and to age- and sex-matched controls without previous clinical history. In total, 5 self-perception measures and 30 RNA expression measures were derived from RA patients and control groups. A correlation analysis based on Spearman correlation and Logistic-regression methods was adopted to assess the correlation between self-perception and RNA expression. Quantitative analysis revealed that RA patients with poor self-perception were closely associated with RNAs expression, In addition, 3 key molecules including AC019117.2, LINC00638, and hsa_circ_0003972 could be used to predict self-perception changes in RA patients. Herein, our results will provide new insights in RA diagnosis however, the underlying mechanisms need to be further explored.

## Introduction

Rheumatoid arthritis (RA) is an inflammatory disease characterized by chronic synovial inflammation and progressive joint destruction (1, 2). The disease has a high morbidity and mortality rate affecting ~1% of the global population (3). In the long-term, RA left untreated, causes disability of body joints hence limits their functions, reduces workability, and more importantly, deteriorates quality of life. In particular, the relationship between self-perception and therapy among self-perception patients has attracted more attention in clinical research (4). Currently, the Disease Activity Score in 28 joints (DAS28), the Visual Analog Scale (VAS), the Self-rating Depression Scale (SDS), the Self-rating Anxiety Scale (SAS) and the MOS 36-Item Short Form Survey (SF-36) have been recommended internationally to evaluate self-perception in RA patients. Therefore, they have been the most accepted evaluation methods by the majority of clinicians (5). A previous study found that 80% of RA patients with depression exhibited a highly significant correlation of depressive symptoms with RA disease activity (6). Further, our previous studies revealed that self-perception was closely related to disease activity indices such as ESR, RF, CRP, IgG in RA patients. Thus, it is suggested that a higher RA disease activity results in poorer self-perception among patients (7, 8).

There is a clear genetic basis to RA but its underlying mechanisms remain unknown (9). Recently, an increasing number of studies have attempted to reveal the important role of epigenetics in the pathogenesis of RA (10, 11). Circular RNAs (circRNAs) and long non-coding RNA (lncRNA), a novel type of endogenous non-coding RNA recently rediscovered (12, 13), are known to modulate the activity of interacting proteins or act as miRNA sponges. Specifically, they competitively associate with miRNAs, which regulate protein-coding gene expression at transcriptional and post-transcriptional levels (14, 15). Previous studies have demonstrated that circRNAs and lncRNAs are involved in regulating the pathogenesis of many diseases. Circular RNA TTBK2 regulates cell proliferation, invasion, and ferroptosis via miR-761/ITGB8 axis in glioma (16) whereas, long non-coding RNA LINC01503 promotes gastric cancer cell proliferation and invasion by regulating Wnt signaling (17). Based on the findings, these RNAs may advance the diagnosis of RA disease in the future. A recent study based on microarray analysis reported that hsa_circ_0000396 and hsa_circ_0130438 are a potential biomarker in the diagnosis of RA (18). Previously, our research showed that hsa_circ_0001200, hsa_circ_0001566, hsa_circ_0003972, and hsa_circ_0008360 are closely related to RA disease severity similar to DAS28 evaluation method (19). However, little information is known about the relationship between self-perception in RA patients and RNA expression.

In this work, we conducted a self-perception questionnaire survey among 30 RA patients and 30 healthy controls. Our main aim was to identify RNAs profiling at peripheral blood mononuclear cells (PBMCs) in RA patients and to explore the relationship between self-perception changes and RNA expression. Notably, based on the findings we identified differentially expressed RNAs in RA patients and the healthy controls via high-throughput sequencing technology, which could be used to predict SPP.

## Materials and Methods

### Clinical Data and Patient Samples

In total, 30 patients diagnosed with RA at the Department of Rheumatology and Immunology in the First Affiliated Hospital of Anhui University of Traditional Chinese Medicine from June 2019 to December 2019 served as RA group. In addition, 30 age- and sex-matched healthy subjects who underwent routine physical examinations in the same hospital during the same period were used as the healthy control group. The healthy controls had no clinical history of tumors, trauma, infectious diseases, or autoimmune diseases. All patients fulfilled the 2010 American College of Rheumatology/European League Against Rheumatism (ACR/EULAR) criteria for RA classification (20). However, patients with malignant tumors, severe liver, and kidney dysfunction, or pregnant women were excluded.

All the study subjects filled in the DAS28, VAS, SAS, SDS, and SF-36 under the guidance of clinical doctors. SF-36 consists of 8 dimensions namely physical functioning (PF), role physical (RP), body pain (BP), general health (GH), vitality (VT), social functioning (SF), role emotional (RE), and mental health (MH).

All clinical measurements were performed by the clinical laboratory staff in our hospital. The clinical laboratory data such as erythrocyte sedimentation rate (ESR), high-sensitivity C-reactive protein (CRP), rheumatoid factor (RF), anti-cyclic citrullinated peptide antibody (CCP), immunoglobulins A (IGA), immunoglobulin G (IGG), immunoglobulin M (IGM), complement 3 (C3), and complement 4 (C4) together with clinical characteristics were determined. This study was approved by the ethics committee from our hospital, and all participating patients provided written informed consent.

### High-Throughput Sequencing and Bioinformatics Analysis

Here 3 individuals (2 females and 1 male, 45–67 years of age) diagnosed with RA following the 2010 American College of Rheumatology (ACR) diagnostic criteria, were selected for RNA-seq. Also, 3 healthy controls (2 females and 1 male, age between 45 and 66 years) without previous clinical history were selected from the Physical Examination Center in our hospital. The 3 RA patients exhibited the active stage of the disease and showed poor self-perception. RNA library construction and RNA sequencing (RNA-seq) were performed via CutSeq Biotech Inc (Shanghai, China). The ribosomal RNA (rRNA) was isolated from total RNA using the Ribo-Zero rRNA Removal Kit (Bacteria) (Illumina) following the manufacturer′s instructions. Sequencing libraries were generated using the TruSeq Stranded emRNA Library Prep Kit (Illumina, Munich, Germany). For quantification and quality checks of the libraries, we used the BioAnalzyer 2100 system and qPCR (Kapa Biosystems, Woburn, MA). The libraries were denatured into single-stranded DNA molecules, captured on Illumina Flow Cells (Illumina), and amplified *in situ* as clusters. Then, the libraries were sequenced for 150 cycles following the manufacturer's instructions.

Elimination of low-quality and adaptor sequences from the raw data was performed with Cutadapt (v1.6). We used EdgeR software (v3.16.5) to normalize the data and extract differentially expressed RNAs. A *P* < 0.05 and absolute fold change ≥ 2.0 signified differentially expressed RNAs. Afterward, the differentially expressed RNAs were selected for GO and KEGG pathway analysis.

### PBMC Preparation and Total RNA Extraction

Exactly 5 mL of whole blood was drawn from RA patients and healthy controls. Thereafter, the PBMCs were isolated through Ficoll–Paque density gradient centrifugation (GE Healthcare, Uppsala, Sweden). The concentration of cells was adjusted to 5–7 × 10^6^ cells per ml and reserved at −80°C until use.

### Validation With Real-Time qPCR

Total RNA from PBMCs of the 60 samples was extracted using Trizol reagent (Invitrogen Life Technologies, Carlsbad, CA, USA) following the manufacturer's instructions. The RNA was then reverse-transcribed to single-stranded cDNA and used as a template to synthesize the second cDNA strand. Aliquots of total RNA samples were used to determine the RNA concentration and purity using the NanoDrop ND-1000 spectral photometer (peqlab). The RNAs were selected based on a combination of *p*-value, fold change, raw intensity, and type. In addition, RNAs with miRNA response elements (MREs) related to RA reported in previous literature were selected preferentially. Eventually, 30 RNAs were selected, including 10 circRNAs and 20 lncRNAs. All qPCR assays were performed via the Viia7 Real-Time PCR System, each sample was replicated three times. Library quality was assessed using the Agilent Bioanalyzer 2100 system. The relative expression levels of RNAs were calculated using the 2^−ΔΔCt^ method. All primer sequences were designed at NCBI database through primer blast in the available sequences of these genes. Primer sequences were summarized and GAPDH was set as internal reference (Table 1).

**Table 1:** Specific RNAs primers used for quantitative RT-qPCR analysis.

**Genename**	**Sequence**
GAPDH	F: GGAGCGAGATCCCTCCAAAAT
	R: GGCTGTTGTCATACTTCTCATGG
hsa_circ_0001200	F: CGGACAGGAGTGAGGAGAAG
	R: TGGCAAGACGCTTGTAACTG
hsa_circ_0001566	F: CATGGAGCTGGATCATGAAA
	R: AGGTTGAGTCTGCCACTTGC
hsa_circ_0003972	F: AGGAAATCACATTCTGCCTGA
	R: CAACGGCTTTGATCACTACG
hsa_circ_0008360	F: TCGAGAACTTTGGGATGGTC
	R: TTTGTGTCTGCGAAGTGCAT
hsa_circ_0000734	F: CAGCTGAAGCAGTTGGAGTG
	R: GATAACGCGGCGGACTATT
hsa_circ_0001402	F: AAACAGCAGCCAAAGGATGT
	R: GCCCATCTTCACAAACTGGT
hsa_circ_0003353	F: TTCCCAGCCTTTTTGAGATG
	R: GGTGGCAACTCCGTATCTGT
hsa_circ_0005732	F: CACACCATCCAATTCCTTCA
	R: TCTGGGGGTCTGTCTTCTTG
hsa_circ_0072428	F: GCACTCAGATGAGGGGAAAG
	R: CTGCATTCAAATCCCCAGAT
hsa_circ_0091685	F: TGCTAACGCACAGTCACACA
	R: ATAAAATGCGGGTCCCTCTC
LINC01504	F:TTGGCTAACGGAGTTTTGCT
	R:CTTCTGAGGCCTGGATCTTG
LINC00968	F:GCCCAGTTGACAGGAAATGT
	R:TTGGTTCTCAATGGGATGGT
FAM95B1	F:GGAGCTCAGTGCCCTCATAG
	R:GCTCCAGGATGATGGTGTCT
MIR503HG	F:CCCCCAACAAAGGAACACTA
	R:ACTTGGGTGGTTTTCAATGC
LINC00304	F:CCGTCCAAGAGCAAAGCTAC
	R:GGCATCAGGCAAAATCAAGT
LINC01146	F:ATTCAGCCAACCAACTGAGG
	R:TCACAGGTTCTGTGGGTCAA
MAPKAPK5-AS1	F: GCGGAAAGTGACCAAGAG
	R: CTTCTCCAGAGCCTGGTCAC
ENST00000619282	F:CCTGGTGGGAGAGAATTGAA
	R: ATGAGAGCCAAGCAAGAGGA
C5orf17	F: CCACACCGACACCTATACCC
	R: TCGACTCTCCACTGTGATGC
LINC01189	F: GTCTGCCCAGCTACTCCAAG
	R: CTCCTACCGCTCCTGTTGAG
LINC01006	F: GTGTGTCAGGCATTGTACCG
	R: GCCCTGTTTCCAAAAGATCA
DSCR9	F: ATTCCCTCCCCTATCACCAG
	R: CCTAGCATACGCTGGAGGAC
MIR22HG	F: TGGAGGAGGGGGTTAGAGTT
	R: GGGGATCACATACCACCTTG
LINC00630	F: GGCTGTTTCGTGAAGGAGAG
	R: AGTAGCCCTGTCTCCAGCAA
LINC00663	F: CCACACTGGTGGATGAGTTG
	R: GCGTGGACATCCAGACTTTT
LINC00638	F: ACAATTCGACCCGTAACAGC
	R: TGCTCGATATTCCCATGTCA
MIAT	F: TGTCTCCATTTGCTCAGTGC
	R: TCAGGATGGTGCACTCTCAG
AC007952.5	F: GGCCAGAAAATGCCTATGAA
	R: GGGATCTCATTCAAGCCAAA
AC019117.2	F: AAGACGGAAAACTCCAAGCA
	R: CTGCAGCTGTGGTCTGAAAG
PSMG3-AS1	F: AGTCTCCTGGGCTACAAGCA
	R: CGGTTCTAGAGGCAAACGAG

### Statistical Analysis

All statistical data were analyzed using SPSS version 23.0 (SPSS, Chicago, IL) statistical package and GraphPad Prism 8.0 (GraphPad; LaJolla, CA). Descriptive data were represented as the mean ± standard deviation for normally distributed variables whereas, data for non-normally distributed variables were represented as median (25–75%). Univariate descriptive statistics were used to describe the sample *X*^2^ tests performed for categorical variables. Further, continuous variables were evaluated using *t*-tests or Mann-Whitney non-parametric tests, as appropriate. Spearman Correlation test and Logistic Regression analysis were adopted for correlation analysis to identify statistically significant RNAs, clinical indices associated with self-perception in RA patients. A *p*-value of *p* < 0.05 was statistically significant (^*^p < 0.05, ^**^p < 0.01, ^***^p < 0.001).

## Results

### General Characteristics of the Study Population

The baseline characteristics of RA patients and healthy controls can be found in Table 2. There were 28 (80%) females with mean age 51.33 (SD = 11.74) years in the RA group, and 28 (80%) females with mean age 51.99 (SD = 8.62) years in the healthy control group. There was no significant difference in mean age and gender between the two groups. Besides, there were no differences in employment, housing or marital status at baseline.

**Table 2:** Baseline characteristics.

**Characteristic**	**RA (*N* = 30)**	**HC (*N* = 30)**	***P*–value**
Age, y, mean (SD)	51.33 (11.74)	51.99 (8.62)	0.963
Female, %	80	80	1.000
BMI, mean (SD)	23.80 (4.51)	24.49 (4.42)	0.972
Ever smoker, %	20	16.67	0.583
Ever drinker, %	23.33	26.67	0.762
Symptom duration, year, median (IQR)^a^	6.00 (2.00, 10.25)	NA	NA
Disease duration, year, median (IQR)^b^	4.5 (1.38, 10.00)	NA	NA
ESR, mm/h, median (IQR)	43.60 (19.00, 59.75)	NA	
CRP mg/L, median (IQR)	8.29 (2.31, 26.54)	NA	
Tender jiont count, 0–28, mean(SD)	9.82 (8.61)	NA	
DAS 28 score, mean (SD)	6.80 (0.94)	NA	
Pain, VAS, 0–100 mm, mean(SD)	66.87 (22.03)	NA	
RF positivity, %	96.67	NA	
CCP positivity, %	86.67	NA	
Presence of radiographic erosions, %	55.33	NA	
Charlson comorbidity Index, mean(SD)	1.29 (1.02)	NA	
Prenisolone use, %	87.72	NA	
**DMARD treatment (at baseline)**			
DMARD-naive, %	60	NA	
MTX monotherapy, %	16.67	NA	
Non-MTX csDMARD, %	6.67	NA	
Combination csDMARD, %	16.67	NA	
**Education status**			
None or primary, %	10	13.33	0.568
Secondary or vocational, %	60	66.67	
Tertiary, %	30	20	
**Housing status**			
Private housing, %	46.67	43.33	1.000
Government housing, %	53.33	56.67	
**Employment status**			
Currently employed, %	33.33	16.67	0.109
Unemployment, retired, or homemaker, %	66.67	83.33	
**Marital status**			
Currently married, %	93.33	96.67	1.000
Single, divorced or widowed, %	6.67	3.33	

*BMI, body mass index; csDMARD, conventional syn-thetic disease-modifying antirheumatic drug; MTX, methotrexate*.

*Data with normal distribution were denoted with mean (SD) deviation, data with skewed distribution were expressed as medians (IQR), and the count data were presented as the number or percentage*.

a *Year from symptom onset to date of first rheumatologist review*.

b *Year from date of diagnosis to date of recruitment to study*.

### Differentially Expressed RNAs in the PBMCs of RA Patients

**Figure 1:**
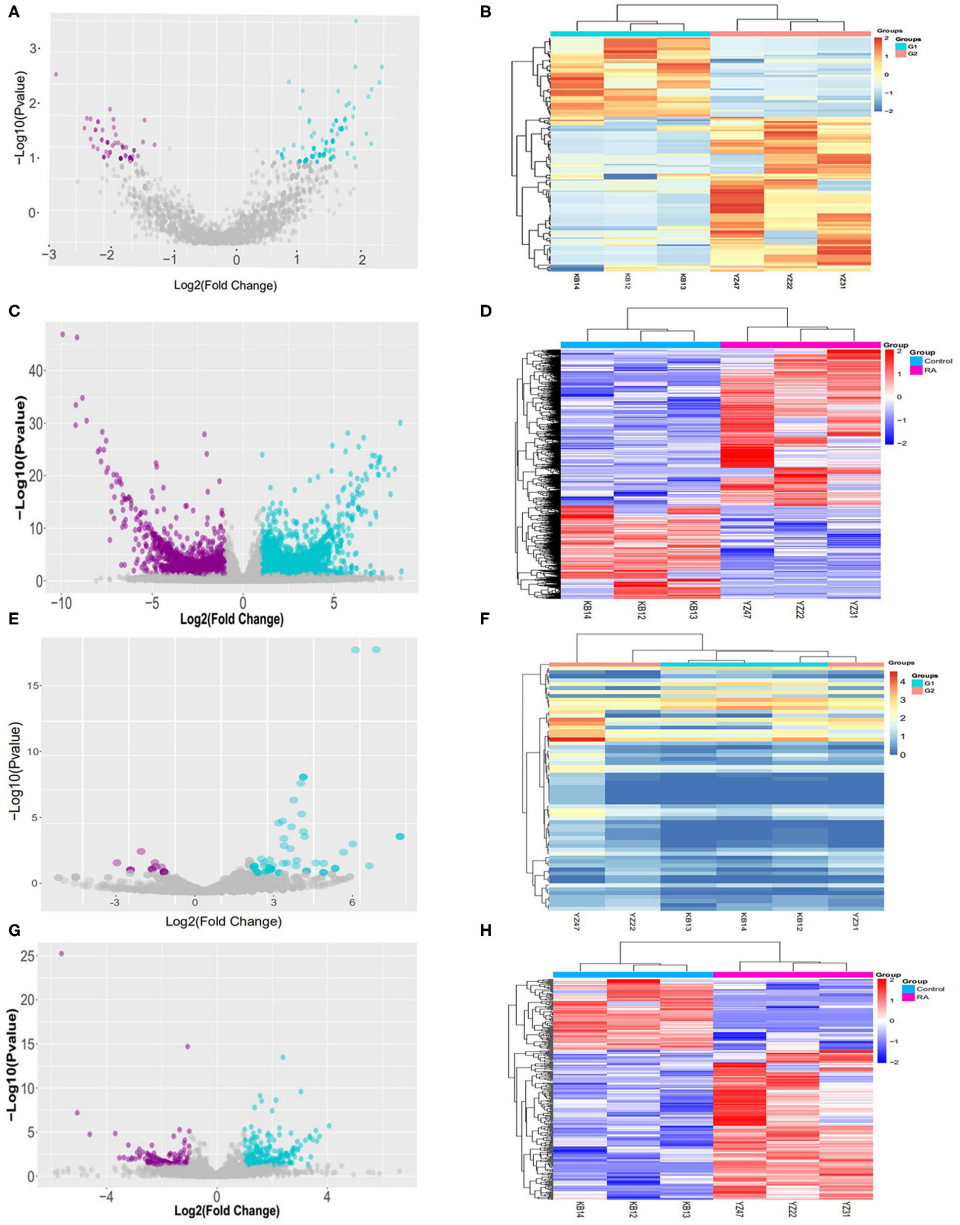
Sequences determining the RNA expression profiles in RA patients and healthy controls. **(A)** Volcano plot of circRNAs. The vertical lines correspond to 1.5-fold up and down while the horizontal line represents a *P*-value of 0.05. **(B)** Heatmap of differentially expressed circRNAs. Red: high relative expression; blue: low relative expression. **(C)** Volcano plot of lncRNAs. The vertical lines correspond to 1.5-fold up and down and the horizontal line represents a *P*-value of 0.05. **(D)** Heatmap of differentially expressed lncRNAs: Red: high relative expression; blue: low relative expression. **(E)** Volcano plot of miRNAs: The vertical lines correspond to 1.5-fold up and down and the horizontal line represents a *P*-value of 0.05. **(F)** Heatmap of differentially expressed miRNAs: Red: high relative expression; blue: low relative expression. **(G)** Volcano plot of mRNAs: The vertical lines correspond to 1.5-fold up and down and the horizontal line represents a *P*-value of 0.05. **(H)** Heatmap of differentially expressed mRNAs: Red: high relative expression; blue: low relative expression.

The differentially expressed RNAs between the RA group and healthy control groups with statistical criteria were determined through fold change and *p*-value (*p*-value < 0.05 absolute fold change ≥2.0). A total of differentially expressed 165 circRNAs, 341 lncRNAs, 63 miRNAs, and 7,895 mRNAs were identified. In contrast to the healthy control group, a total of 109 circRNAs from the RA group were significantly upregulated, and 56 significantly downregulated as shown by a volcano plot (Figure 1A) and a cluster heatmap (Figure 1B). A total of 231 lncRNAs were significantly upregulated, and 110 were significantly downregulated in the RA group as shown by a volcano plot (Figure 1C) and a clustered heatmap (Figure 1D). In the RA group, 52 miRNAs were markedly upregulated, whereas 11 miRNAs were significantly downregulated as depicted in the volcano plot (Figure 1E) and cluster heatmap (Figure 1F). In total, 4,916 mRNAs were strikingly upregulated, and 2,934 significantly downregulated in the RA group as shown by a volcano plot (Figure 1G) and a cluster heatmap (Figure 1H).

### Function of Differentially Expressed circRNAs and mRNAs in RA Patients

**Figure 2:**
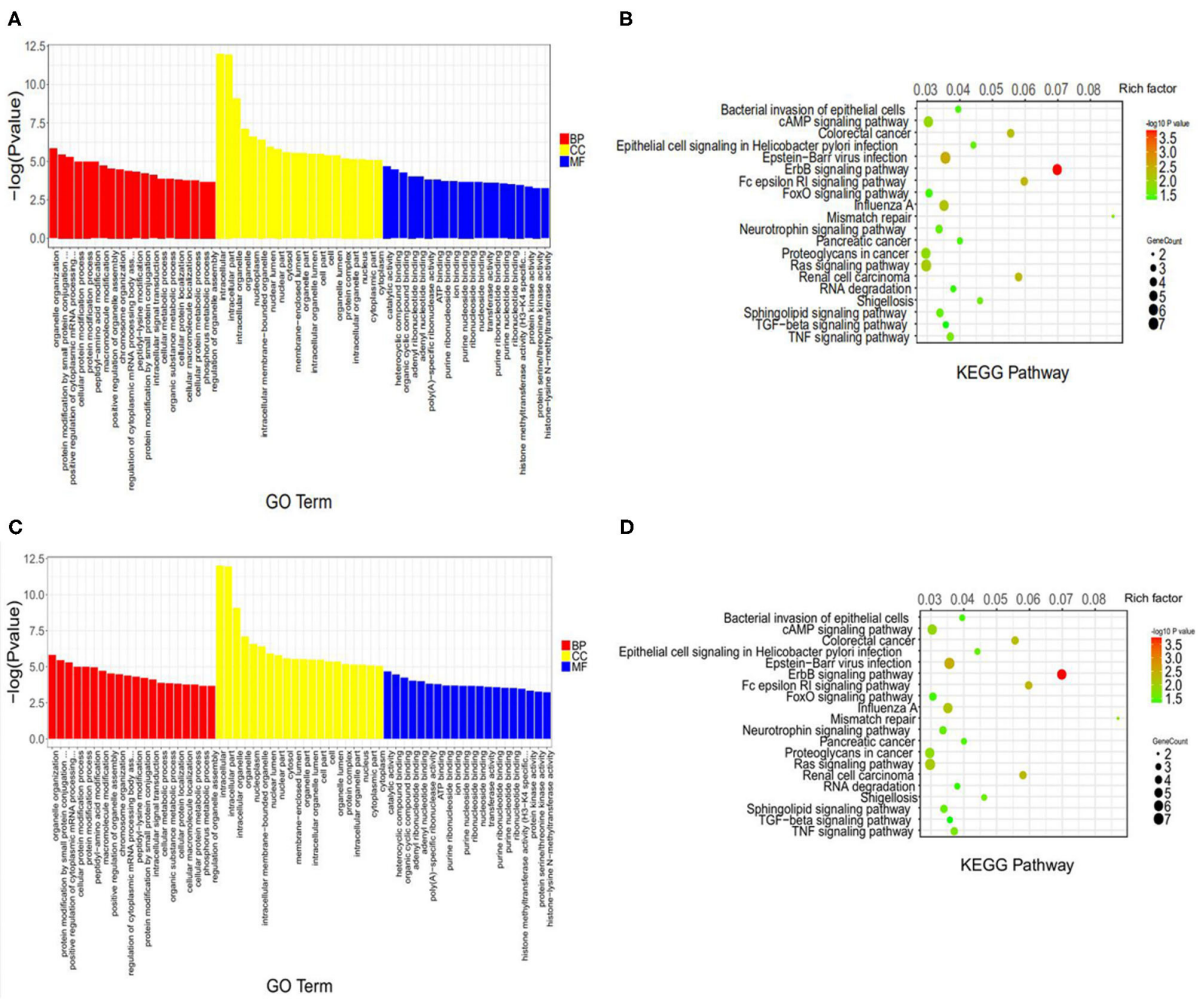
GO analysis and KEGG pathway annotation of differentially expressed RNAs**. (A,C)** The top 20 significant changes in GO biological process classification of circRNAs and mRNAs. Red, yellow, and blue bars represent biological Process, cellular component, and molecular function, respectively. **(B,D)** The top 20 significant changes in the KEGG pathway classification of circRNAs and mRNAs. The bar plot shows the top Enrichment Score [–log10 (*P*-value)] of the significant pathway. The color represents the level of differences, a darker color indicates a greater difference. The circle represents the relationship.

To explore the differentially expressed circRNAs and mRNAs, we performed Gene Ontology (GO) terms, Kyoto Encyclopedia of Genes and Genomes (KEGG), and functional enrichment analyses. The results revealed that the differentially expressed circRNAs were associated with the top two enriched biological processes including organelle organization and positive regulation of cytoplasmic mRNA processing. The top two enriched cellular components include intracellular and intracellular components whereas the top two enriched molecular functions are catalytic activity and heterocyclic compound binding (Figure 2A). The analysis results showed that the differentially expressed mRNAs contained the top two enriched biological processes: organelle organization and protein modification by small protein conjugation, the top two enriched cellular component: intracellular and intracellular part, the top two enriched molecular function: cytoplasm and catalytic activity (Figure 2C). Furthermore, the top 20 pathways connected with circRNAs and mRNAs function in the RA group were defined by the KEGG analysis. Both bacterial invasion of epithelial cells and the cAMP signaling pathway were the top two in circRNAs and mRNAs functions (Figures 2B,D).

### Basic Characteristics of Selected RNAs

A total of 30 differentially expressed genes were screened in the RA group (Table 3). Out of these, 10 differentially expressed circRNAs including 5 upregulated and 5 downregulated genes were selected. However, 20 differentially expressed lncRNAs including 5 upregulated and 15 downregulated genes were selected (Table 3).

**Table 3:** Basic characteristics of the 30 differently expressed RNAs in RA patients.

**RNAs**	**Position**	***p***	**Fold change**	**Regulation**	**Gene symble**
Hsa_circ_0001200	chr21:46275124-46281186	0.0146	2.26	Up-regulation	PTTG1IP
Hsa_circ_0001566	chr5:179688683-179707608	0.0146	2.26	Up-regulation	MAPK9
Hsa_circ_0003972	chr9:96238537-96261168	0.0239	2.10	Up-regulation	FAM120A
Hsa_circ_0003353	chr2:188348850-188368497	0.0484	1.61	Up-regulation	TFPI
Hsa_circ_0091685	chrX:149895686-149901202	0.0456	−1.54	Up-regulation	MTMR1
Hsa_circ_0008360	chr22:41277773-41278181	0.0168	−2.16	Down-regulation	XPNPEP3
Hsa_circ_0000734	chr17:1746096-1756483	0.0197	−1.80	Down-regulation	RPA1
Hsa_circ_0001402	chr4:38091552-38104778	0.0116	−1.28	Down-regulation	TBC1D1
Hsa_circ_0005732	chr2:157406119-157414094	0.0263	−1.95	Down-regulation	GPD2
Hsa_circ_0072428	chr5:49698119-49707217	0.0456	−1.54	Down-regulation	EMB
LINC00968	chr8:56520378-56559823	0.0014	2.75	Up-regulation	PENK
MIR503HG	chrX:134543448-134546759	0.0029	2.53	Up-regulation	HPRT1
MIR22HG	chr17:1713448-1717174	0.031	1.66	Up-regulation	SMYD4
ENST00000619282	chr12:121190868-121191518	0.000	1.80	Up-regulation	P2RX7
AC019117.2	ENST00000658415.1	0.0169	1.90	Up-regulation	MFSD9
LINC00630	100287765	0.0152	1.21	Up-regulation	EPM2A
MAPKAPK5-AS1	chr12:111839768-111842902	0.022	−2.50	Down-regulation	TMEM116
LINC01504	chr9:72305017-72315464	0.0003	−3.21	Down-regulation	GDA
C5orf17	chr5:23951348-24178263	0.028	−2.01	Down-regulation	NBPF14
LINC01189	chr9:62452490-62522017	0.044	−2.16	Down-regulation	ACSL1
LINC01006	chr7:156638366-156640654	0.030	−2.48	Down-regulation	RNF32
DSCR9	chr21:37208503-37221740	0.039	−1.94	Down-regulation	TTC3
FAM95B1	chr9:40323571-40329218	0.0025	−2.54	Down-regulation	GDA
LINC00663	284440	0.0332	−1.13	Down-regulation	EPM2A
LINC00638	196872	0.0182	−2.09	Down-regulation	GIMAP7
MIAT	440823	0.0287	−1.34	Down-regulation	Miat
AC007952.5	ENST00000572818.2	0.0240	−2.52	Down-regulation	USP34
LINC01146	chr14:88024539-88084749	0.0041	−2.48	Down-regulation	GALC
PSMG3-AS1	114796	0.0319	−1.74	Down-regulation	MFSD9
LINC00304	chr16:89155925-89163598	0.0040	−2.79	Down-regulation	ZNF778

### Verification of Selected RNAs

**Figure 3:**
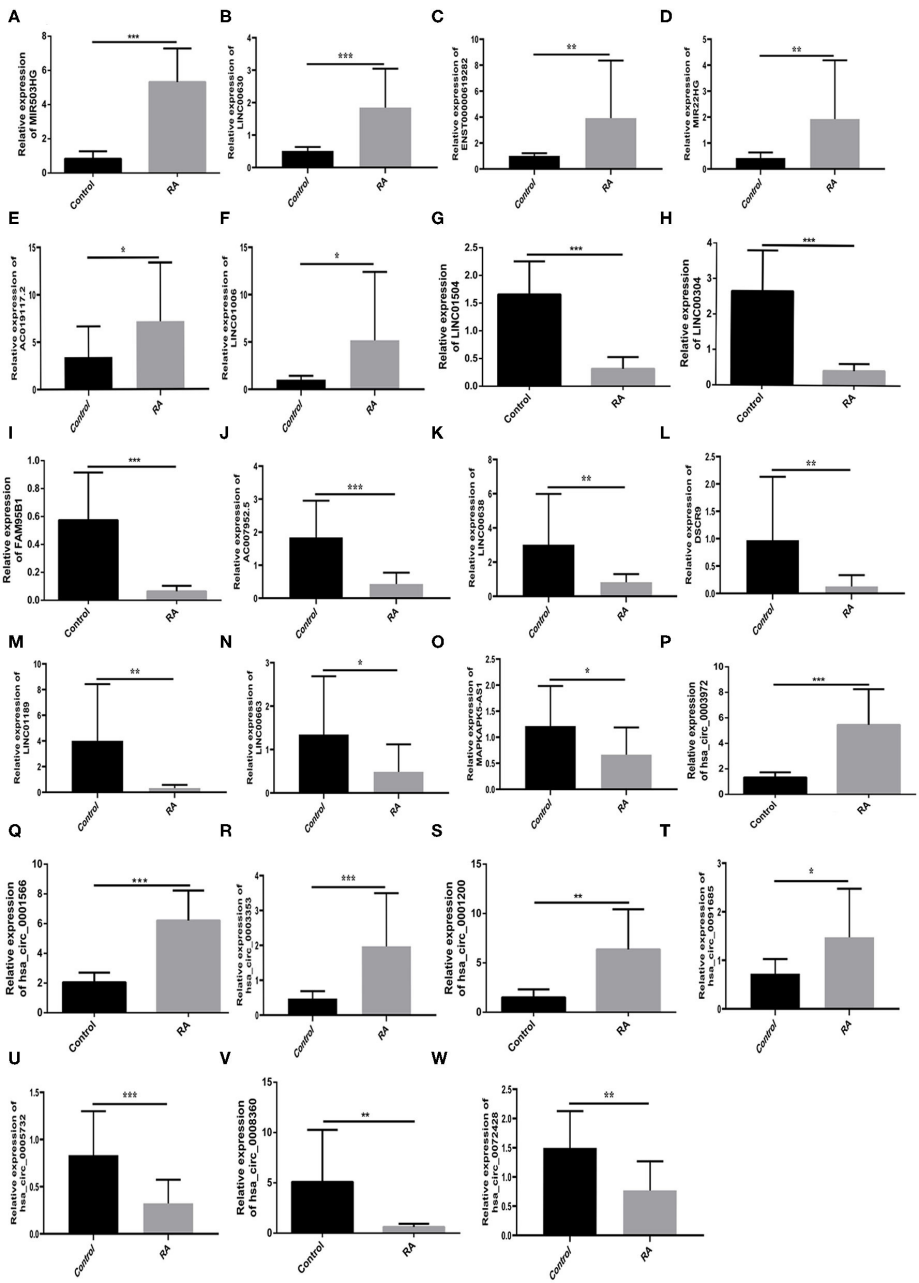
Quantitative RT-qPCR determines the relative expression levels of RNAs. **(A–F,P–T)** MIR503HG, LINC00630, hsa_circ_0001566, hsa_circ_0003972, hsa_circ_0003353 (*p* < 0.001), MIR22HG, ENST00000619282, hsa_circ_0001200 (*p* < 0.01), LINC01006, AC019117.2, hsa_circ_0091685 (*p* < 0.05) exhibited the same increasing trend as the sequencing results. **(G–O,U–W)** LINC00304, LINC01504, FAM95B1, AC007952.5, hsa_circ_0005732 (*p* < 0.001), LINC01189, DSCR9, LINC00638, hsa_circ_0008360, hsa_circ_0072428 (*p* < 0.01), MAPKAPK5-AS1, LINC00663 (*p* < 0.05) exhibited the same decreasing trend as the sequencing results. *p < 0.05, **p < 0.01, and ***p < 0.001 vs. control.

PBMCs from 30 RA patients and 30 healthy controls were used for verification through RT-qPCR. On the base of sequencing, we selected 30 RNAs from the most significant RNAs for further verification. Among the selected 30 RNAs, the expression levels of MIR503HG, LINC00630, hsa_circ_0001566, hsa_circ_0003972, hsa_circ_0003353 (*p* < 0.001), MIR22HG, ENST00000619282, hsa_circ_0001200 (*p* < 0.01), LINC01006, AC019117.2, hsa_circ_0091685 (*p* < 0.05) were significantly high in the RA group than in the healthy group (Figures 3A–F, P–T). Additionally, the expression levels of LINC00304, LINC01504, FAM95B1, AC007952.5, hsa_circ_0005732 (*p* < 0.001), LINC01189, DSCR9, LINC00638, hsa_circ_0008360, hsa_circ_0072428 (*p* < 0.01), MAPKAPK5-AS1, LINC00663 (*p* < 0.05) were significantly low in the RA group compared to the healthy group (Figures 3G–O,U–W).

### Spearman Correlation Analysis of Self-Perception, RNA Expression, and Clinical Indices in RA Patients

**Figure 4:**
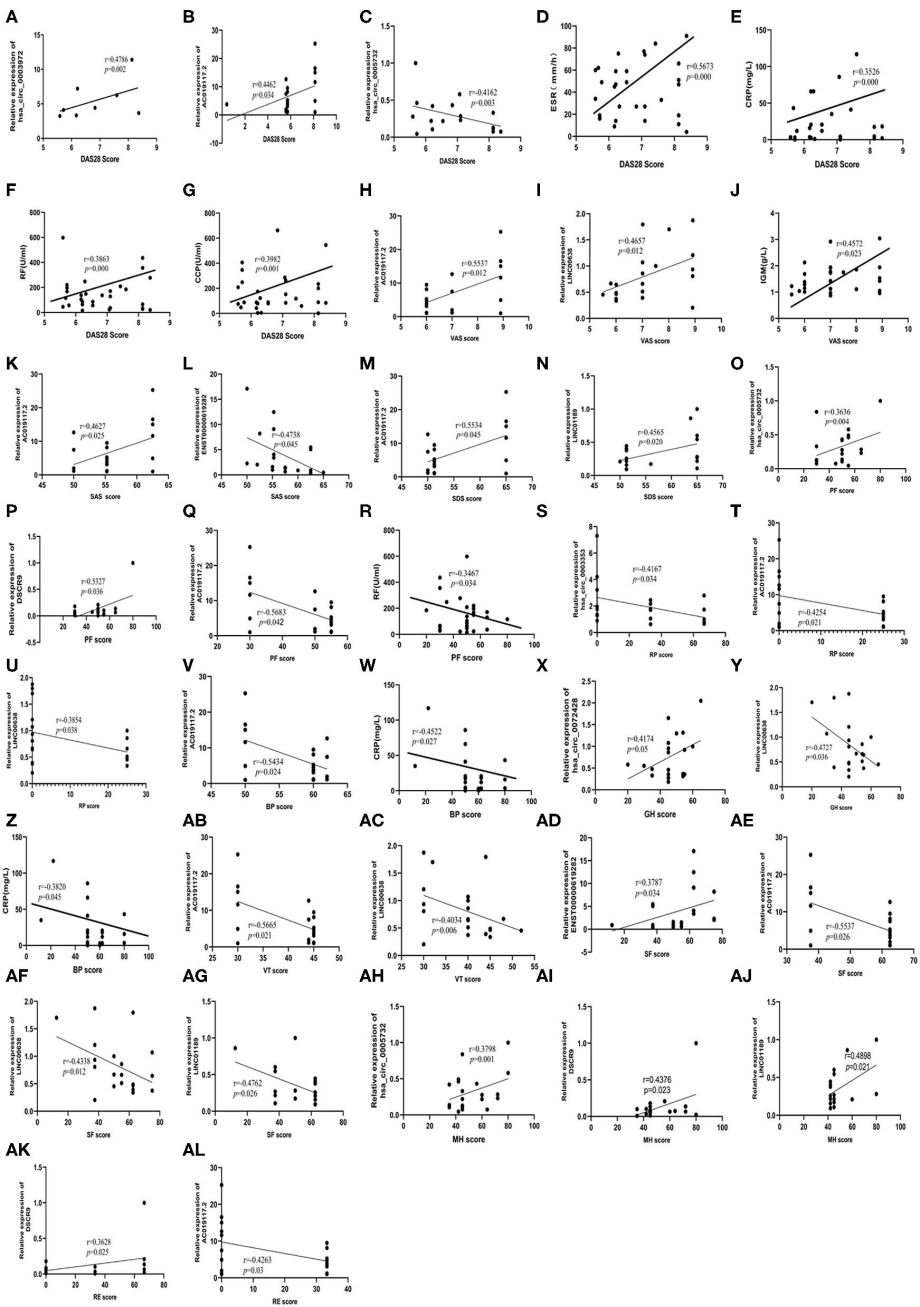
Correlation between SPP and RNAs, clinical indexes. **(A–G)** DAS28 score correlated positively with hsa_circ_0003972, hsa_circ_0005732, AC019117.2, and correlated negatively with ESR, CRP, RF, and CCP. **(H–J)** VAS score correlated positively with LINC00638, AC019117.2, and IGM. **(K,L)** SAS score correlated positively with AC019117.2 and correlated negatively with ENST00000619282. **(M,N)** SDS score correlated positively with AC019117.2, LINC01189. **(O–R)** PF score correlated positively with hsa_circ_0005732, DSCR9, and correlated negatively with AC019117.2, RF. **(S–U)** RP score correlated negatively with hsa_circ_0003353, LINC00638, AC019117.2. **(V,W)** BP score positively correlated with AC019117.2 and correlated negatively with CRP. **(X–Z)** GH score positively correlated with hsa_circ_0072428, a negatively correlated with LINC00638, CRP. **(AB,AC)** VT score negatively correlated with AC019117.2, LINC00638. **(AD–AG)** SF score correlated positively with ENST00000619282, AC019117.2, LINC00638, and negatively correlated with LINC01189. **(AH–AJ)** MH score correlated positively with hsa_circ_0005732, LINC01189, DSCR9. **(AK,AL)** RE score positively correlated with DSCR9, and correlated negatively with AC019117.2.

We analyzed the relationship between the expression of 30 validated RNAs, which reflects the quality of life, and clinical indices which reflect the severity of the disease was analyzed. We found that DAS28 correlated positively with hsa_circ_0003972 and hsa_circ_0005732 and AC019117.2, while correlated negatively with ESR, CRP, RF, CCP (Figures 4A–G). In addition, VAS correlated positively with LINC00638 AC019117.2, and IGM (Figures 4H–J). SAS positively correlated with AC019117.2 and negatively with ENST00000619282 (Figures 4K,L). SDS correlated positively with AC019117.2 and LINC01189 (Figures 4M,N). Furthermore, PF correlated positively with hsa_circ_0005732, DSCR9, and negatively with AC019117.2 RF (Figures 4O–R). RP correlated negatively with hsa_circ_0003353, LINC00638, and AC019117.2 (Figures 4S–U). BP correlated positively with AC019117.2 and negatively with CRP (Figures 4V,W). GH correlated positively with hsa_circ_0072428, negatively with LINC00638, CRP (Figures 4X–Z). VT correlated negatively with AC019117.2 and LINC00638 (Figures 4AB,AC). Moreover, SF correlated positively with ENST00000619282, AC019117.2, and LINC00638, negatively with LINC01189 (Figures 4AD–AG). MH correlated positively with hsa_circ_0005732, LINC01189, DSCR9 (Figures 4AH–AJ) whereas RE correlated positively with DSCR9 and negatively correlated with AC019117.2 (Figures 4AK,AL).

### Logistic Regression Analysis of Self-Perception Related Factors

Logistic Regression analysis of risk factors in self-perception among RA patients was carried out. Significant difference in DAS28 were found between RA patients with hsa_circ_0003972 (Wald Chi-square value = 15.782, potence ratio: 2.891, *P* = 0.009), AC019117.2 (Wald Chi-square value = 9.930, potence ratio: 3.782, *P* = 0.021),ENST00000619282 (Wald Chi-square value = 3.349, potence ratio: 3.821, *P* = 0.027),DSCR9 (Wald Chi-square value = 12.341, potence ratio: 5.673, *P* = 0.034),ESR (Wald Chi-square value = 8.563, potence ratio: 4.672, *P* = 0.002),indicating that these RNAs are risk factors for DAS28. Therefore, higher RNA expression levels implied a higher DAS28 score (Table 4). Additionally, we found a significant difference in the VAS score in RA patients with LINC00638 (Wald Chi-square value = 3.441, potence ratio: 1.243, *P* = 0.026) and AC019117.2 (Wald Chi-square value = 4.893, potence ratio: 2.784, *P* = 0.038), indicating that they are risk factors for VAS. Hence, the higher the RNAs expression, the higher the VAS score (Table 5). Furthermore, a significant difference in SAS was found in RA patients with hsa_circ_0003353 (Wald Chi-square value = 4.628, potence ratio: 2.753, *P* = 0.034), MAPKAPK5-AS1 (Wald Chi-square value = 3.572, potence ratio: 1.563, *P* = 0.047), indicating that these are risk factors for SAS. This implies that the higher the RNAs expression, the higher the SAS score (Table 6). In addition, significant differences in GH were found in RA patients with hsa_circ_0072428 (Wald Chi-square value = 7.523, potence ratio: 2.754, *P* = 0.012), indicating that hsa_circ_0072428 is a risk factor for GH, thus, the higher RNA expression, the lower the GH score (Table 7).

**Table 4:** Logistic Regression analysis of DAS28 related factors.

**Variable**	**Coefficient**	**Standard deviation**	**Wald Chi-square value**	**Degree of freedom**	***P*-value**	**Potence ratio**	**95% confidence interval**	
							**Lower limit**	**Upper limit**
hsa_circ_0003972	O.672	0.738	15.782	1	0.009	2.891	0.334	3.678
AC019117.2	0.772	0.733	9.930	1	0.021	3.782	0.672	4.672
ENST00000619282	0.478	0.356	3.349	1	0.027	3.821	2.894	8.783
DSCR9	0.674	0.839	12.341	1	0.034	5.673	2.236	7.678
ESR (mm/h)	0.672	0.682	8.563	1	0.002	4.672	0.560	3.798

**Table 5:** Logistic Regression analysis of VAS related factors.

**Variable**	**Coefficient**	**Standard deviation**	**Wald Chi-square value**	**Degree of freedom**	***P*-value**	**Potence ratio**	**95% confidence interval**	
							**Lower limit**	**Upper limit**
LINC00638	0.684	0.798	3.441	1	0.026	1.243	0.563	3.782
AC019117.2	0.841	0.673	4.893	1	0.038	2.784	0.633	2.344

**Table 6:** Logistic Regression analysis of SAS related factors.

**Variable**	**Coefficient**	**Standard deviation**	**Wald Chi-square value**	**Degree of freedom**	***P*-value**	**Potence ratio**	**95% confidence interval**	
							**Lower limit**	**Upper limit**
hsa_circ_0003353	0.683	0.945	4.628	1	0.034	2.753	0.672	4.893
MAPKAPK5-AS1	0.563	0.639	3.572	1	0.047	1.563	0.722	3.849

**Table 7:** Logistic Regression analysis of GH related factors.

**Variable**	**Coefficient**	**Standard deviation**	**Wald Chi-square value**	**Degree of freedom**	***P*-value**	**Potence ratio**	**95% confidence interval**	
							**Lower limit**	**Upper limit**
hsa_circ_0072428	0.675	0.745	7.523	1	0.012	2.754	1.783	6.420

## Discussion

In this study, we filtered 30 RNAs with differential expression that were validated by RT-qPCR technique. The results showed that 27 exhibited the same trends as the sequencing results. Through Spearman correlation analysis we demonstrated that 9 out of 27 genes had a significant correlation with self-perception in RA patients. Further Logistic Regression analysis of RNA expression revealed that. AC019117.2, LINC00638, hsa_circ_0003972 were the strong risk factors for self-perception. Besides, the key RNAs associated with self-perception isolated from 3 RA patients and 3 healthy people were selected and verified for RNA-sequencing. Other studies currently being conducted have identified some specific RNAs from RA patients which are associated with self-perception and clinical indicators.

RA is an autoimmune disease associated with increased depression and decreased quality of life (21, 22). Notably, immune mechanisms hold the dominant position for enhancing the possibility of RA occurrence and changes in self-perception (6). However, there is a need to identify new biomarkers and explore their functions since the details of the RA mechanisms remain ambiguous. Both CircRNAs and lncRNAs function by competing ceRNAs, also known as miRNA “sponges,” which are RNA transcripts that competitively for the binding to specific miRNAs (23). For instance, decreased hsa_circ_0044235 in PBMCs has been confirmed to have a significant efficacy in the diagnosis of RA (24). Elsewhere, a study revealed that the expression of LncRNA ENST00000456270 is strongly associated with the serum levels of IL-6, TNF-a, and the Simplified Disease Activity Index (SDAI) of the RA patient (25). In addition, FOXM1/LINC00152 feedback loop regulates the proliferation and apoptosis in rheumatoid arthritis fibroblast-like synoviocytes via Wnt/β-catenin signaling pathway (26). However, no study has analyzed the correlation between RNA expression, self-perception, and clinical indices. Several studies have observed a strong link between the disease activity and quality of life amongst RA patients, however, its development mechanisms from RNA expression perspective have not been pronounced (4). Consequently, the roles of RNAs in patients with poor self-perception are unclear. As a result, it is essential to profile RNAs expression and discover new biomarkers, which will provide a new awareness for self-perception changes in RA patients.

In the present study, we recruited 30 RA patients and 30 age- and sex-matched healthy participants who acted as controls. All of the participants filled in the DAS28, VAS, SAS, SDS, SF-36 under the guidance of clinical doctors. The results uncovered poor self-perception in RA patients compared to the healthy controls. Moreover, DAS28, VAS, SAS, SDS, ESR, CRP were significantly increased (*P* < 0.05), whereas PF, RP, BP, GH, VT, SF, RE, MH were significantly decreased (*P* < 0.05). Meanwhile, RF, CCP, IGA, IGG, IGM, C3, C4 were significantly high compared to the healthy control group (*P* < 0.05). We identified 3 RA patients and 3 healthy control for RNA-seq. Of note, we identified differentially expressed 165 circRNAs, 341 lncRNAs, 63 miRNAs, and 7,895 mRNAs, respectively. Besides, GO enrichment and KEGG analysis suggested that RNAs are involved in protein modification, catalytic activity, and cAMP signaling pathways. Furthermore, we increased the number of samples and carried out RT-qPCR to verify sequencing results. Subsequently, we identified 30 RNAs based on their expression distribution in each specimen. As a consequence, 11 RNAs in the RA group were significantly higher than in the healthy group whereas 12 RNAs in the RA group were significantly lower than in the healthy group. Our results provide insights into the pathomechanism of RA and a theoretical basis for the in-depth exploration of the RNAs function in RA.

Based on the results from Spearman correlation and Logistic Regression analyses, we proudly report that 3 RNAs were closely related to RA patients with poor self-perception. The 3 RNAs include AC019117.2, LINC00638, and hsa_circ_0003972. Further, Spearman correlation analysis indicated that DAS28 correlated positively with AC019117.2, hsa_circ_0003972, whereas VAS correlated positively with LINC00638. Also, Logistic Regression analysis suggested that AC019117.2 and hsa_circ_0003972 are risk factors for DAS28 and LINC00638 is a risk factor for VAS.

Of note, AC019117.2, LINC00638, and hsa_circ_0003972 may provide a new theoretical basis for research on RNAs, hence promoting the use of these RNAs as valuable biomarkers in RA patients with poor self-perception. Despite laying the key foundation in the understanding of RA pathogenesis, this study had some limitations. For instance, the potential mechanisms of the key RNAs in RA patients with poor self-perception still needs to be further elucidated. Furthermore, it is possible that the sample size was too small to detect a difference between seropositive and seronegative RA patients. Additionally, there are still some few RNAs with minimal relationship with self-perception thus deserves further exploration. In the future, the function of other differentially expressed RNAs should be confirmed in a larger sample size. Further *in vitro* and animal studies should be conducted to advance the comprehension of the detailed mechanisms and specific functions of RNAs in RA patients with poor self-perception.

In summary, AC019117.2, LINC00638, and hsa_circ_0003972 are potentially significant predictors in RA patients with poor self-perception. However, the mechanisms of these RNAs need to be further investigated.

## Data Availability Statement

The datasets presented in this study can be found in online repositories. The names of the repository/repositories and accession number(s) can be found in the article/supplementary material.

## Ethics Statement

The studies involving human participants were reviewed and approved by The Ethics Committee of the First Affiliated Hospital of Anhui University of Traditional Chinese Medicine. The patients/participants provided their written informed consent to participate in this study. Written informed consent was obtained from the individual(s) for the publication of any potentially identifiable images or data included in this article.

## Author Contributions

JWe, JL, BW, HJ, LW, LX, and YuS contributed to the study design. JWe contributed to data analysis, wrote the first draft, and revised the manuscript. YaS, YZ, XD, XW, and JWa contributed to the questionnaire survey on patients, specimens, and data collection. JL and BW supervised the project and helped revise the manuscript. All authors reviewed and accepted the content of the final manuscript. All authors contributed to the article and approved the submitted version.

## Conflict of Interest

The authors declare that the research was conducted in the absence of any commercial or financial relationships that could be construed as a potential conflict of interest.
